# Supplementary material for: Overexpression of a Cytosolic 6-Phosphogluconate Dehydrogenase Gene Enhances the Resistance of Rice to Nilaparvata lugens
Source: Plants (Basel). 2020 Nov 10;9(11):1529. doi: 10.3390/plants9111529 (PMC7696191; doi:10.3390/plants9111529)
Supplement: Supplementary file 1 [file plants-09-01529-s001.pdf]

Supplementary Materials:

Overexpression of a Cytosolic 6-Phosphogluconate Dehydrogenase Gene Enhances the Resistance of Rice to *Nilaparvata lugens*

Lin Chen, Peng Kuai, Miaofen Ye, Shuxing Zhou, Jing Lu, Yonggen Lou *

State Key Laboratory of Rice Biology & Ministry of Agriculture Key Lab of Molecular Biology of Crop Pathogens and Insects, Institute of Insect Sciences, Zhejiang University, Hangzhou 310058, China; chenlin88@126.com (L.C.); kpchen7493@163.com (P.K.); 11616070@zju.edu.cn (M.Y.); 3090100232@zju.edu.cn (S.Z.); jing_lu@zju.edu.cn (J.L.)

* Correspondence: Prof. Yonggen LOU, yglou@zju.edu.cn; Tel.: (86)-0571-8898-2622

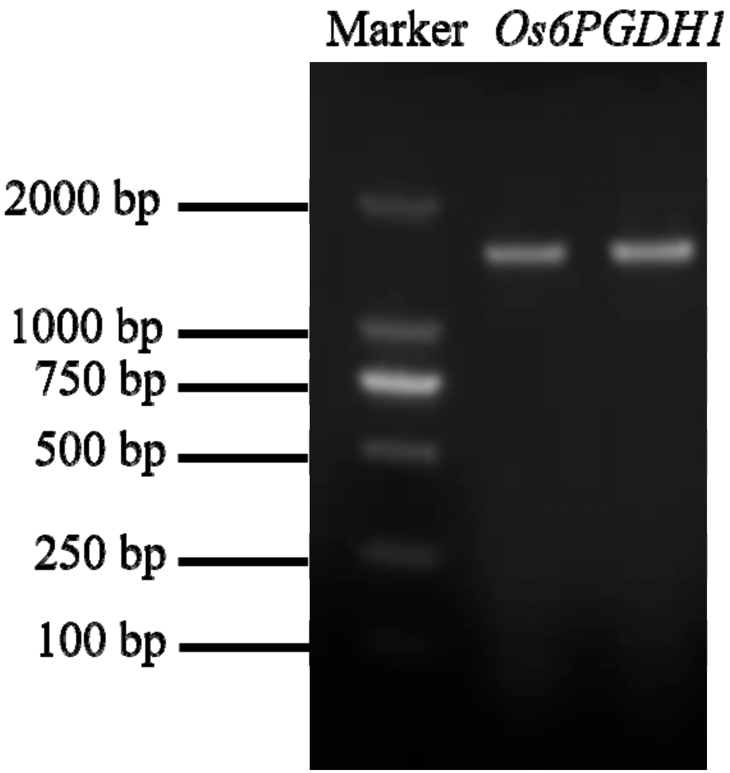

Figure S1. Gel electrophoresis of PCR amplified full-length segments of *Os6PGDH1* from cDNA library of Xiushui 110. The PCR product is about 1500 bp.

```
1     ATGGCTGTCACTAGAATTGGTCTTGCTGGCCTTGCGGTCATGGGGCAGAACCTTGCCCTCAACATTGCAGAGAAA
1      M  A  V  T  R  I  G  L  A  G  L  A  V  M  G  Q  N  L  A  L  N  I  A  E  K
76    GGGTTCCCTATCTCTGTCTACAACAGGACGACTTCTAAGGTTGATGAGACCGTTCAGCGCGCCAAGGTAGAAGGA
26     G  F  P  I  S  V  Y  N  R  T  T  S  K  V  D  E  T  V  Q  R  A  K  V  E  G
151   AACCTTCCTGTGTACGGGTTCCATGACCCTGCATCCTTTGTGAACTCCATTCAGAAGCCACGTGTTGTCATCATG
51     N  L  P  V  Y  G  F  H  D  P  A  S  F  V  N  S  I  Q  K  P  R  V  V  I  M
226   CTTGTCAAGGCTGGTGCACCAGTGGACCAGACCATTGCAACTCTTGCAGCACACTTGGAGCAGGGTGACTGTATT
76     L  V  K  A  G  A  P  V  D  Q  T  I  A  T  L  A  A  H  L  E  Q  G  D  C  I
301   ATTGATGGAGGAAATGAGTGGTACGAGAACACTGAGAGGAGGGAGAAGGCAATGGAGGAGCGTGGCCTCCTCTAT
101    I  D  G  G  N  E  W  Y  E  N  T  E  R  R  E  K  A  M  E  E  R  G  L  L  Y
376   CTTGGGATGGGTGTTTCCGGAGGAGAGGAGGGTGCCCGCAATGGCCCGTCCTTGATGCCTGGTGGCTCATTCGAG
126    L  G  M  G  V  S  G  G  E  E  G  A  R  N  G  P  S  L  M  P  G  G  S  F  E
451   GCGTACAAGTACATTGAAGATATTCTTCTCAAGGTGGCTGCCCAGGTTCCTGATAGTGGCCCGTGCGTCACATAC
151    A  Y  K  Y  I  E  D  I  L  L  K  V  A  A  Q  V  P  D  S  G  P  C  V  T  Y
526   ATTGGCAAAGGTGGATCTGGAAACTTTGTCAAGATGGTTCACAACGGAATTGAGTATGGTGACATGCAACTGATT
176    I  G  K  G  G  S  G  N  F  V  K  M  V  H  N  G  I  E  Y  G  D  M  Q  L  I
601   TCTGAGGCATATGATGTTCTCAAGTCAGTTGGTAAGCTCACAAACAGTGAGCTGCAGCAGGTGTTTTCTGAGTGG
201    S  E  A  Y  D  V  L  K  S  V  G  K  L  T  N  S  E  L  Q  Q  V  F  S  E  W
676   AACAAGGGTGAGCTCCTCAGTTTCCTGATTGAGATCACAGCCGACATATTTAGCATCAAGGATGACCAGGGTTCA
226    N  K  G  E  L  L  S  F  L  I  E  I  T  A  D  I  F  S  I  K  D  D  Q  G  S
751   GGCCACCTGGTCGACAAGGTCCTGGACAAGACTGGGATGAAGGGAACTGGGAAGTGGACTGTGCAGCAGGCCGCA
251    G  H  L  V  D  K  V  L  D  K  T  G  M  K  G  T  G  K  W  T  V  Q  Q  A  A
826   GAGCTTTCTGTGGCTGCTCCTACAATTGAGGCATCCTTGGATTCCAGGTTCCTCAGTGGGTTGAAGGATGAGCGT
276    E  L  S  V  A  A  P  T  I  E  A  S  L  D  S  R  F  L  S  G  L  K  D  E  R
901   GTTGAGGCTGCCAAGGTCTTCCAAGGTGACTTCTCCAGCAACTTGCCAGTGGACAAGGCACAGCTGATTGAAGAC
301    V  E  A  A  K  V  F  Q  G  D  F  S  S  N  L  P  V  D  K  A  Q  L  I  E  D
976   GTGAGGCAGGCTCTTTACGCCTCGAAGATCTGCAGCTACGCTCAGGGCATGAACATCATCAAGGCCAAGAGCATG
326    V  R  Q  A  L  Y  A  S  K  I  C  S  Y  A  Q  G  M  N  I  I  K  A  K  S  M
1051  GAGAAAGGGTGGAGCCTCAACCTCGGTGAGCTAGCGAGGATCTGGAAGGGAGGGTGCATCATCCGTGCCATCTTC
351    E  K  G  W  S  L  N  L  G  E  L  A  R  I  W  K  G  G  C  I  I  R  A  I  F
1126  CTGGACCGCATCAAGAAGGCCTACGACAGGAACTCCGACCTCGCCAACCTGCTCGTGGACCCTGAGTTCGCTCAG
376    L  D  R  I  K  K  A  Y  D  R  N  S  D  L  A  N  L  L  V  D  P  E  F  A  Q
1201  GAGATCATGGACAGGCAAGCGGCATGGCGCAGGGTGGTCTGCCTTGCCATCAACAATGGCGTCAGCACCCCAGGC
401    E  I  M  D  R  Q  A  A  W  R  R  V  V  C  L  A  I  N  N  G  V  S  T  P  G
1276  ATGTCGGCGAGTCTCGCGTACTTCGACTCCTACCGGAGGGACAGGCTGCCTGCCAACCTGGTGCAGGCACAGAGG
426    M  S  A  S  L  A  Y  F  D  S  Y  R  R  D  R  L  P  A  N  L  V  Q  A  Q  R
1351  GACTATTTCGGGGCTCACACCTACGAGAGGGTCGACATGCCCGGCTCCTTCCACACCGAGTGGTTCAAGATCGCG
451    D  Y  F  G  A  H  T  Y  E  R  V  D  M  P  G  S  F  H  T  E  W  F  K  I  A
1426  CGTGCTGCTAAGATGTGA
476    R  A  A  K  M  *
```

Figure S2. The nucleotide and deduced amino acid sequence of Os6PGDH1. The stop codon is marked by an asterisk.

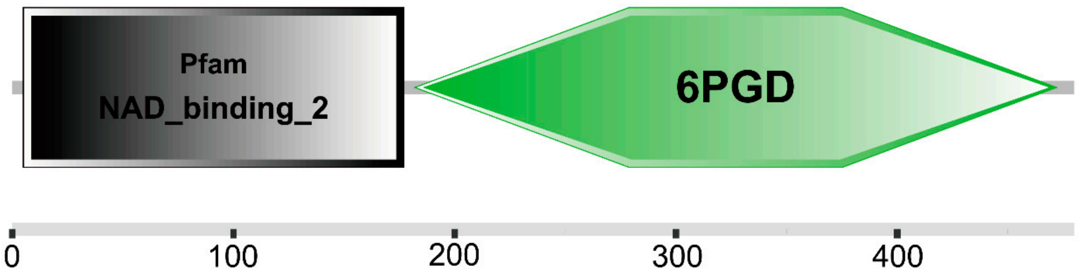

Figure S3. Prediction of the conserved domains of Os6PGDH1. The nicotinamide adenine dinucleotide phosphate (NADP)-binding site (NAD_binding_2) and 6-phosphogluconate (6PD) binding site (6PGD) are indicated by black and green, respectively.

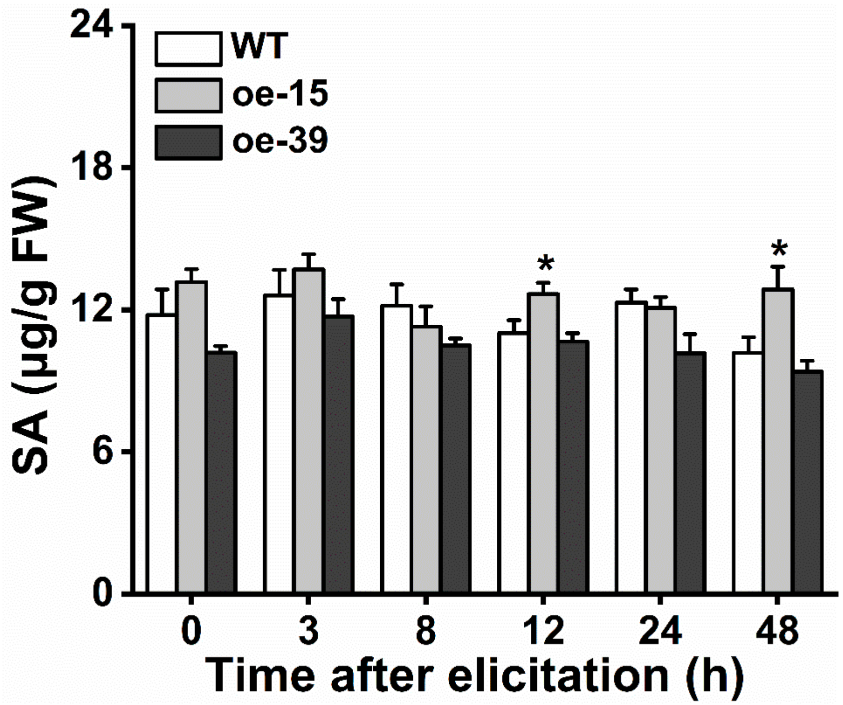

Figure S4. Levels of SA in oe6PGDH lines and WT plants at indicated time points after they were individually infested by 15 gravid BPH females. Values are means + SE, n = 6. Asterisks indicate significant differences in oe6PGDH lines compared with WT plants (one-way ANOVA followed by Duncan's multiple range test, P < 0.05).

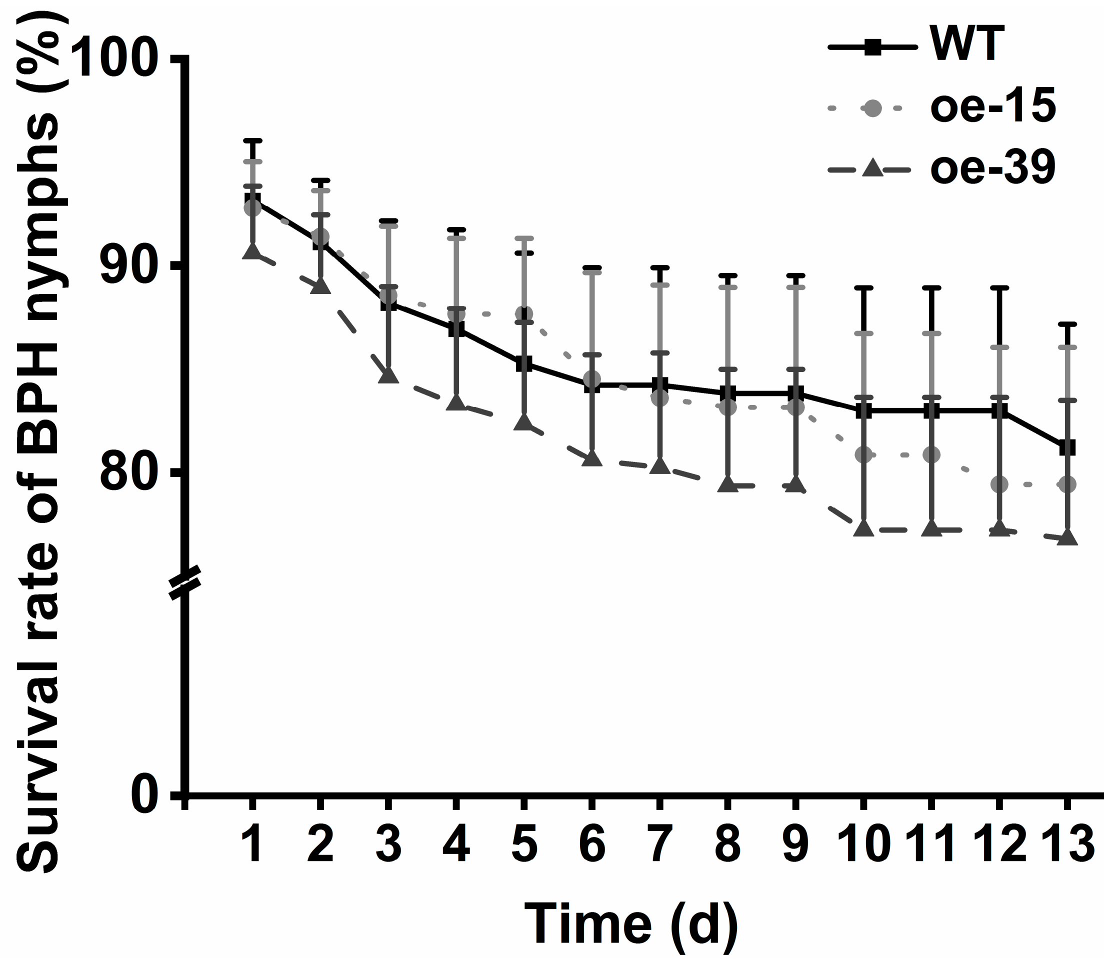

Figure S5. The survival rate of 15 newly hatched BPH nymphs that fed on oe6PGDH or WT plants. Values are means + SE, n = 13. Asterisks indicate significant differences in oe6PGDH lines compared with WT plants (one-way ANOVA followed by Duncan's multiple range test, $P < 0.05$).

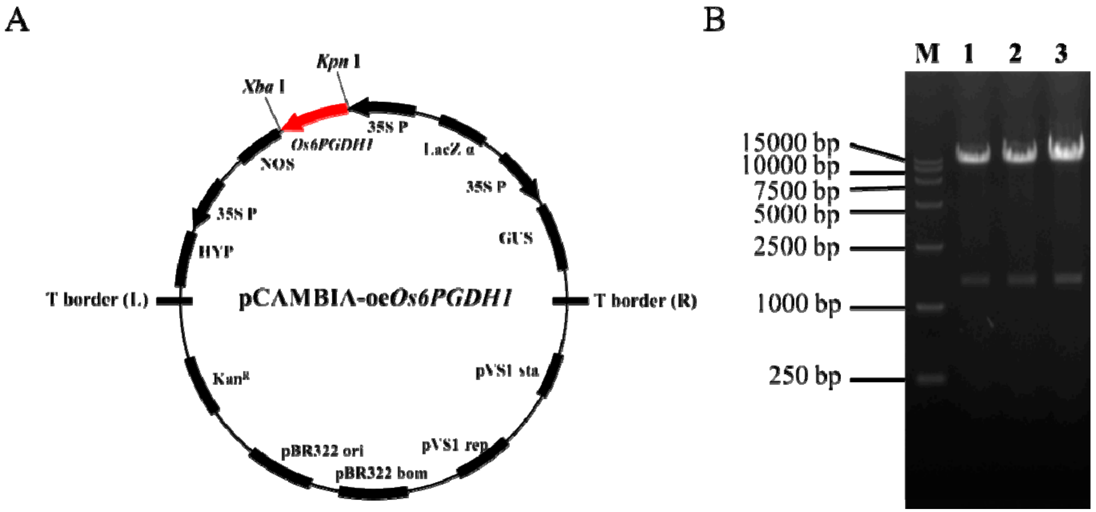

Figure S6. Construction of *Os6PGDH1*-overexpression vector used for rice transformation. A, Vector construction of pCAMBIA-*oeOs6PGDH1*; B, restriction digestion of pCAMBIA-*oeOs6PGDH1* with *Kpn* I and *Xba* I.

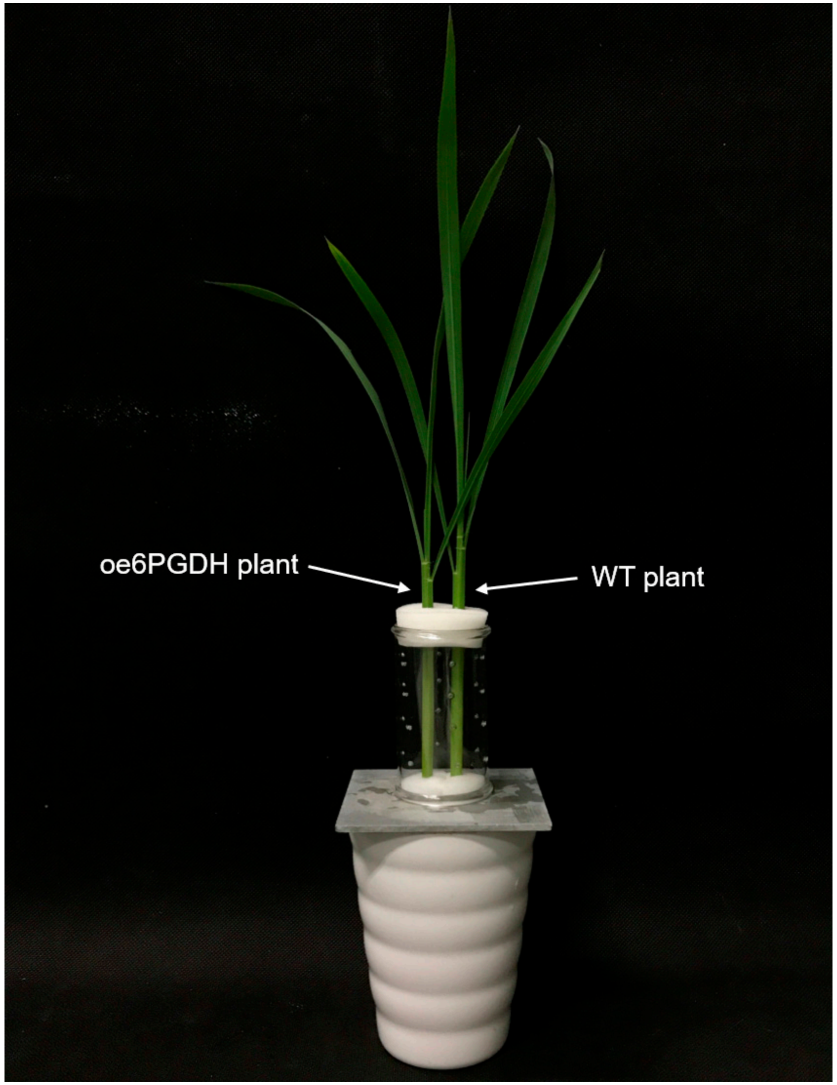

Figure S7. The experimental setup of BPH bioassay for feeding and oviposition preference.

Table S1. Primers and probes used for qRT-PCR of target genes

Gene name	TIGR ID	Forward primer (5′-…-3′)	Reverse primer (5′-…-3′)	Probe (5′-…-3′)
OsACTIN	Os03g50885	TGGACAGGTTATCACCATTGGT	CCGCAGCTTCCATTCCTATG	HEX-CGTTTCCGCTGCCCTGAGGTCC-BHQ1
Os6PGDH1	Os06g02144	GGAACTGGGAAGTGGACTGT	TTGCTGGAGAAGTCACCTTG	FAM-TGGCAGCCTCAACACGCTCA-BHQ1
